# Beer–Lambert law for optical tissue diagnostics: current state of the art and the main limitations

**DOI:** 10.1117/1.JBO.26.10.100901

**Published:** 2021-10-28

**Authors:** Ilze Oshina, Janis Spigulis

**Affiliations:** University of Latvia, Institute of Atomic Physics and Spectroscopy, Biophotonics Laboratory, Riga, Latvia

**Keywords:** tissue absorption, optical scattering, Beer’s law

## Abstract

**Significance:** Beer–Lambert law (BLL) is a widely used tool for contact and remote determination of absorber concentration in various media, including living tissues. Originally proposed in the 18th century as a simple exponential expression, it has survived numerous modifications and updates. The basic assumptions of this law may not be fulfilled in real measurement conditions. This can lead to mistaken or misinterpreted results. In particular, the effects to be additionally taken into account in the tissue measurements include anisotropy, scattering, fluorescence, chemical equilibria, interference, dichroism, spectral bandwidth disagreements, stray radiation, and instrumental effects.

**Aim:** We review the current state of the art and the main limitations of remote tissue diagnostics using the BLL. Historical development of updating this law by taking into account specific additional factors such as light scattering and photon pathlengths in diffuse reflectance is described, along with highlighting the main risks to be considered by interpreting the measured data.

**Approach:** Literature data related to extension and modification of the BLL related to tissue assessment and concentration estimation of specific tissue molecules are collected and analyzed. The main emphasis here is put on the optical measurements of living tissue chromophore concentrations and estimation of physiological parameters, e.g., blood oxygen saturation.

**Results:** Modified expressions of the BLL suitable for several specific cases of living tissue characterization are presented and discussed.

**Conclusions:** Applications of updated/modified Beer–Lambert law (MBLL) with respect to particular measurement conditions are helpful for obtaining more reliable data on the target tissue physiological state and biochemical content. MBLL accounting for the role of scattering in several ways appears to be a successful approach. Extended MBLL and BLL in the time domain form could provide more accurate results, but this requires more time resources to be spent.

## 1 Introduction

Beer–Lambert law (BLL) describes how attenuation of light relates to the properties of the medium through which it travels; it is referred also to as Beer–Lambert–Bouguer law or simply Beer’s law.

This law is widely applied in biomedical optics, for example, to calculate oxygen saturation in human tissues,^1^^,^^2^ to determine the molar absorbance of bilirubin in blood plasma sample,^3^ and to determine the concentration of hemoglobin components^4^ or optical pathlength (OPL) through a tissue.^5^

BLL is so popular mainly because it can be easily integrated in the calculation system, and the calculation time is short thanks to the linear relationship between the measured light attenuation and the medium’s absorbance. Besides, it requires less computational power as compared to other models, such as diffusion approximation,^6^^,^^7^ Monte Carlo simulations,^8^^–^^10^ hyperspectral imaging,^11^ or artificial neural networks.^12^ Dispersion analysis can be used as an alternative, which then allows not only determination of the dispersion parameters but also the concentration of an analyte directly from the transmittance and reflectance spectra.^13^

BLL assumes that the radiation beam is monochromatic, collimated and oriented orthogonally to the sample surface; it is absorbed by single molecular species independently of others; interaction between molecules does not change probability of absorption; the sample is homogeneous and does not scatter the radiation.^14^ However, in reality strict observation of all these conditions may appear impossible, especially if measurements of a living tissue are taken. Therefore, various extensions and modifications of BLL have been proposed to avoid errors in the measured data and misinterpretation of the obtained results.

This paper is intended as a review where historical and more recent literature data related to development of BLL with complementary modifications are collected. The main emphasis is put on BLL applicability for the concentration measurements of living tissue chromophores (e.g., blood hemoglobin, bilirubin) and estimation of physiological parameters, e.g., blood oxygen saturation. Origins of the BLL concept and its developments/improvements over the time will be discussed, along with the main limitations related to BLL applications for human tissue measurements and diagnostics.

## 2 History and Updates of BLL

### 2.1 Origins of the Beer–Lambert–Bouguer Law

#### 2.1.1 Bouguer’s law

Pierre Bouguer, called also the father of photometry, performed experiments measuring and comparing visually perceived brightness of different objects. In his paper of 1729,^15^ he stated Bouger’s law: in a medium of uniform transparency the light remaining in a collimated beam is an exponential function of the length of the path in the medium.

Bouguer’s law is valid if the absorbing medium is homogenous and does not scatter radiation. The incident radiation should be monochromatic, or at least within a bandwidth narrower than that of the medium absorption band. Incident radiation must be collimated, i.e., consist of parallel rays traveling the same length within the chosen medium. As only the remaining light is considered, this law ignores the attenuation mechanism and is also valid for turbid media to the extent that multiple scattering is negligible.

#### 2.1.2 Lambert’s law

Johann Heinrich Lambert in his paper of 1760^16^ described Bouguer’s statement mathematically. Lambert’s law states: the absorbance A and light pathlength d are directly proportional in a homogenous medium while the intensity of the transmitted radiation decreases with increasing thickness of the absorbing medium:

$$I=I_{0}e^{-\mu_{a}d},$$ (1)

$$A=\log(\frac{I_{0}}{I})\propto d,$$ (2)

where $I_{0}$ is the intensity of the incident beam, I is the intensity of the beam after transmission through a thickness d (cm), and $\mu_{a}$ is the absorption coefficient (${\mathrm{cm}}^{-1}$) of the medium.

#### 2.1.3 Beer’s law

Beer^17^ extended this exponential absorption law in 1852 to incorporate the concentration of solutions (mass of absorbing material) in the absorption coefficient. Beer’s law states that absorbance of monochromatic light in a homogeneous (transparent) medium via it travels through is directly proportional to the concentration of the sample substance c:

A∝c. (3)

#### 2.1.4 Beer–Lambert–Bouguer law

**Fig. 1:**
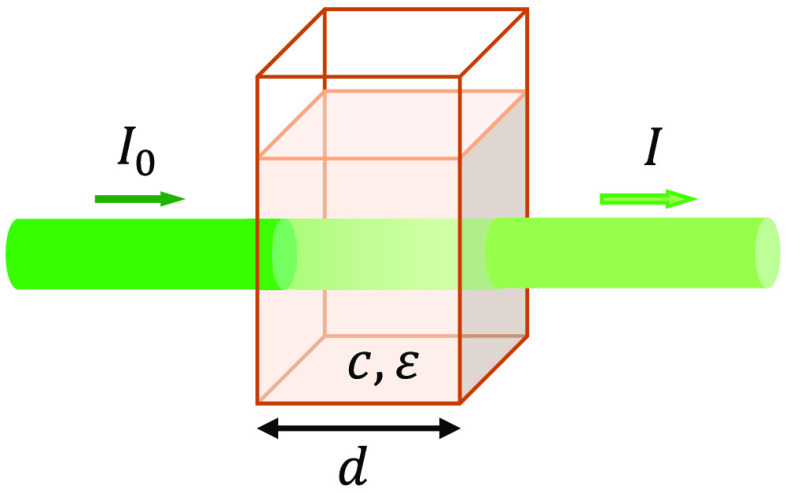
Illustration of the Beer–Lambert–Bouguer law. $I_{0}$, intensity of the incident beam; I, intensity of the beam after transmission through a medium of thickness d; c, concentration; ε, extinction coefficient.

Combining Lambert and Beer law we get the Beer–Lambert–Bouguer Law (Fig. 1), usually called BLL or simply Beer’s law: light absorbed by a substance dissolved in a fully transmitting infinitesimally thin solvent is directly proportional to the concentration of the substance and the pathlength of the light through the solution:

$$A(\lambda )=\log(\frac{I_{0}}{I})=\varepsilon (\lambda )\cdot d\cdot c,$$ (4)

where A is absorbance, ε is the extinction coefficient in ${\mathrm{cm}}^{-1}\;M^{-1}$, d is the thickness of the medium in cm, and c is the molar concentration in M. The constant ε also goes by the names “absorptivity,” “molar absorption coefficient,” “absorbancy index,” and “attenuation coefficient.” Extinction coefficient is used for decimal logarithm units; attenuation coefficient, for natural logarithm units.

BLL can be considered as an extension of Bouguer’s law to solutions of fixed thickness and with variable concentration of the absorbing solute.

### 2.2 Modifications of the Beer–Lambert Law for Tissue Diagnostics

#### 2.2.1 Corrections due to light scattering by blood

Blood is one of the main light absorber and diffuser in living tissues, e.g., in skin. Therefore, it must highly be taken into account when using Beer–Lambert law.

Twersky analyzed absorption of human blood and introduced the idea of supplementing the BLL with light intensity loss due to scattering from the red blood cells:^18^

$$OD=\log(\frac{I_{0}}{I})=\varepsilon cd-\log(10^{-sH(1-H)d}+\frac{q_{\alpha }}{q}(1-10^{-sH(1-H)d})),$$ (5)

where OD is optical density (OD measurements, compared to absorbance, take both absorption and scattering into consideration); ε is the extinction coefficient; c is the average concentration of absorbing molecules (mM/l); d is the slab thickness for transmittance measurements or OPL for reflectance measurements (cm); $s=2Lk^{2}{\left(\eta -1\right)}^{2}$ is a factor depending on wavelength, particle size, and orientation; L is length factor determined by the dimensions of the particles; $k=\frac{2\pi }{\lambda }$ is the wave number; $\eta =\frac{n_{Hb}}{n_{suspending\;medium}}$ is the relative refractive index (if η=1, there is no scattering); H is haematocrit; and q is a factor depending on light detection efficiency. With this additional equation part “$-\log(10^{-sH(1-H)d}+\frac{q_{\alpha }}{q}(1-10^{-sH(1-H)d}))$” calculations for human blood measurements are significantly more reliable compared to the original law. It also gives opportunities to analyze wider range of samples.

Anderson et al.^19^ rewrote it in a shorter form as

OD=mε+B, (6)

$$m=slope=cd\;,\;B=y-intercept=-\log(10^{-sH(1-H)d}+\frac{q_{\alpha }}{q}(1-10^{-sH(1-H)d})).$$ (7)

If ε for hemoglobin is high, then absorption dominates and OD has linear dependency on concentration. If ε is small, then scattering dominates and OD has parabolic concentration dependency. This form can be easily used for blood oxygen saturation calculation (see Sec. 4.1.).

One significant impact on blood absorption must be considered: the shielding effect.^20^ It outlines problems with blood vessels of larger diameters. In this case effective light absorption is reduced because light is less absorbed in the inner region of these vessels, and therefore, reflection is higher. This effect is not as significant for smaller blood vessels within the same tissue volume.

#### 2.2.2 Corrections for tissue diffuse reflectance

Delpy et al.^5^ presented modified BLL (MBLL) for tissue diffuse reflectance as

$$OD=-\log(\frac{I}{I_{0}})=DPF\cdot \mu_{a}d_{io}+G,$$ (8)

where DPF is differential pathlength factor, dependent upon the absorption and scattering coefficients ($\mu_{a}$ and $\mu_{s}$) and the scattering phase function; and $d_{io}$ is inter-optode distance between the light source and detector; G is a geometry dependent factor. Since G is usually unknown, the absolute values for chromophore concentration cannot be determined using Eq. (8). The smallest value for d is determined as

$$d_{\min}=\frac{v_{c}\cdot t}{n},$$ (9)

where $v_{c}$ is the speed of light in vacuum, t is photon transit time, and n is refractive index of the medium. This method opens up opportunities to estimate some parameters, like OPL through tissues, in case of a scattering medium.

DPF values for biological tissues usually are in the range from 3 (muscle) to 6 (adult head).^21^ In semi-infinite geometry, DPF is only negligibly dependent on $d_{io}$ if $d_{io}\sqrt{3\mu_{a}{\mu_{s}}^{\prime }}\gg 1$ ($d_{io}$ is the inter-optode distance between the light source and detector, $\mu_{s}^{\prime }$ is the reduced scattering coefficient, $\mu_{s}^{\prime }=\mu_{s}(1-g)$, and g is the anisotropy factor);^21^^,^^22^ for soft tissues the condition is $d_{io}>2.5\;\mathrm{cm}$. DPF experimental values were calculated from time of flight measurements in reflection mode.^21^

Analytical formula of the DPF for human forehead was presented by Scholkmann et al.^23^ It depends on wavelength (λ,nm), age (Age, years), and tissue type:

$$\mathrm{DPF}(\lambda ,\text{Age})=223.3+0.05624\cdot {\text{Age}}^{0.8493}-5.723\cdot 10^{-7}\cdot \lambda^{3}+0.001245\cdot \lambda^{2}-0.9025\cdot \lambda .$$ (10)

It is assumed that scattering impact is high and it changes negligibly during the measurement, therefore DPF is constant at a certain wavelength; the medium is assumed to be homogeneous.

MBLL in an integral form^24^ is presented as

$$A^{\prime }(\mu_{a})=\int_{0}^{\mu_{a}}\langle L\rangle ({\mu_{a}}^{\prime })d{\mu_{a}}^{\prime }+A^{\prime }(\mu_{a}=0),$$ (11)

where $A^{\prime }(\mu_{a})$ is the total attenuation of the medium, ⟨L⟩=DPF/d is the mean pathlength of detected photons, and $A^{\prime }(\mu_{a}=0)$ is attenuation due to scattering. This microscopic form of BLL showed promising results comparing with time- and frequency-domain measurements.

More accurate expression of MBLL where L is replaced by its average value over the range of absorption coefficient from zero to $\mu_{a}$ is proposed in Ref. 25, as follows:

$$A^{\prime }(\mu_{a})=\mu_{a}\langle \overset{¯}{L}\rangle (\mu_{a})+A^{\prime }(\mu_{a}=0),$$ (12)

where $\langle \overset{¯}{L}\rangle (\mu_{a})=\frac{1}{\mu_{a}}\int_{0}^{\mu_{a}}\langle L\rangle d{\mu_{a}}^{\prime }$ is mean average pathlength of the detected back-scattered photons.

Change in the attenuation can be expressed as

$$\delta A^{\prime }(\mu_{a})=\langle \overset{¯}{L}\rangle \delta \mu_{a}+\mu_{a}\delta \langle \overset{¯}{L}\rangle ,$$ (13)

where

$$\mu_{a}\delta \langle \overset{¯}{L}\rangle =\mu_{a}[\frac{\left\langle \overset{¯}{L}\rangle (\mu_{a}\right)}{\mu_{a}}-\frac{1}{\mu_{a}^{2}}\int_{0}^{\mu_{a}}\langle L\rangle (\mu_{a}^{\prime })d\mu_{a}^{\prime }]\delta \mu_{a}.$$ (14)

The differential form of the MBLL (dMBLL) is correct for relatively small changes of attenuation, if G (geometry-dependent factor) is constant and the absorption is homogeneous.^26^

Measurements are taken either at two different states of the tissues or at two different wavelengths.

In the first case, for example, one state during the brain monitoring is when person is walking ($I_{1}$) and another when standing still ($I_{2}$):

$$\Delta A=\ln\;\frac{I_{1}}{I_{2}}=L\Delta \mu_{a},$$ (15)

$$\Delta \mu_{a}(\lambda )=\varepsilon_{O_{2}Hb}(\lambda )\Delta c_{O_{2}Hb}+\varepsilon_{HHb}(\lambda )\Delta c_{HHb},$$ (16)

where $O_{2}Hb$ is oxyhemoglobin and HHb is deoxyhemoglobin.

Another approach for determination of $\Delta c_{O_{2}Hb}$ and $\Delta c_{HHb}$ is to measure attenuation changes at two wavelengths, $\lambda_{1}$ and $\lambda_{2}$, for dual-wavelength measurement:

$$\Delta c_{O_{2}Hb}=\frac{\varepsilon_{HHb}^{\lambda_{1}}\frac{\Delta A^{\lambda_{2}}}{L^{\lambda_{2}}}-\varepsilon_{HHb}^{\lambda_{2}}\frac{\Delta A^{\lambda_{1}}}{L^{\lambda_{1}}}}{\varepsilon_{HHb}^{\lambda_{1}}\varepsilon_{O_{2}Hb}^{\lambda_{2}}-\varepsilon_{HHb}^{\lambda_{2}}\varepsilon_{O_{2}Hb}^{\lambda_{1}}},$$ (17)

$$\Delta c_{HHb}=\frac{\varepsilon_{O_{2}Hb}^{\lambda_{1}}\frac{\Delta A^{\lambda_{2}}}{L^{\lambda_{2}}}-\varepsilon_{O_{2}Hb}^{\lambda_{2}}\frac{\Delta A^{\lambda_{1}}}{L^{\lambda_{1}}}}{\varepsilon_{O_{2}Hb}^{\lambda_{1}}\varepsilon_{HHb}^{\lambda_{2}}-\varepsilon_{O_{2}Hb}^{\lambda_{2}}\varepsilon_{HHb}^{\lambda_{1}}}.$$ (18)

#### 2.2.3 Extended modified Lambert–Beer model

Huong et al.^27^ presented extended modified Lambert–Beer model (EMLB) to allow a more accurate prediction of the oxygen saturation $SO_{2}$ and carbon monoxide saturation SCO in the skin blood.

MBLL expressed as

$$OD(\lambda )=G_{0}+\mu_{a}d_{0}$$ (19)

has been extended in the following way (EMLB):

$$OD(\lambda )=G_{0}+\mu_{a}d_{0}+G_{1}\lambda +\lambda e^{-\mu_{a}d_{1}},$$ (20)

where $\mu_{a}$ is light absorption in dermis, $d_{0}$ is light pathlength, $G_{1}\lambda$ is light attenuation due to scattering process and absorption in epidermis, and the exponent is used to express light scattering in dermis. With a reference to results of Monte Carlo simulations for the two-layered skin model, data calculated accordingly to Eqs. (19) and (20) were mutually compared. Calculated mean absolute error with MBLL was 10%, and with extended MBLL it was 0.4%, showing that extended MBLL is more precise but also it requires additional computer resources.

**Fig. 2:**
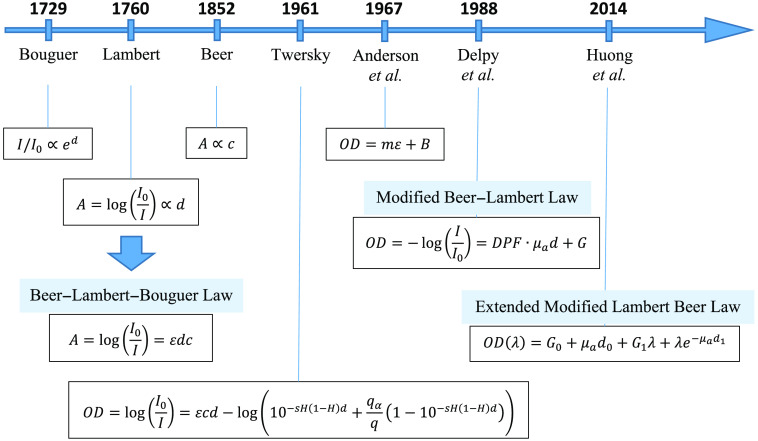
Historical development of the BLL updates.

Historical development of BLL is shown in Fig. 2.

#### 2.2.4 Corrections accounting penetration depth in the skin

Barbosa et al.^28^ in their work tried to determine light penetration depth into the tissues at three wavelengths (660, 830, and 904 nm). Subjects: rat skin, pig skin, lipids, and muscles.

BLL for each wavelength λ:

$$I(\lambda )=I_{0}(\lambda )e^{-\mu d},$$ (21)

where I is transmitted intensity, $I_{0}$ is laser incident intensity, d is slab thickness, μ is the attenuation coefficient, and $\mu =\mu_{a}+\mu_{s}$.

Reflectance for the air–tissue (first) and tissue–air (second) interfaces was taken into account as:

$$R={\left(\frac{n_{t}-n_{i}}{n_{t}+n_{i}}\right)}^{2},$$ (22)

where n is the refraction index, t is transmitted, i is incident.

Tissue attenuation coefficient:

$$\mu =-\frac{1}{d}[\ln(\frac{I_{tm}}{I_{im}})-\ln{\left(1-R^{2}\right)}^{2}],$$ (23)

where $I_{tm}$ is the measured transmitted intensity, $I_{im}$ is the measured incident intensity, and d is tissue slab thickness.

Light penetration depth into the skin was calculated as inverse value of the attenuation coefficient. This simple form of BLL failed only at higher absorber or scatterer concentrations, because the relation between attenuation and absorbance is not linear anymore. Regardless, for samples with small concentrations it is possible to use simple methods with less computational resources, which are just as reliable.^28^

#### 2.2.5 Beer–Lambert law in the time domain form

BLL is applied also with respect to x-rays and gamma-rays in the time domain form:^29^

$$T_{x}=\frac{N_{f}\cdot T_{0}}{N_{0}}\cdot e^{-\mu \cdot d},$$ (24)

where $T_{x}$ is time measurement interval, $N_{f}$ is number of photons (can be chosen as the minimum value of counts measured), $T_{0}$ is the time interval during which the radioactive source emitted initial number of photons $N_{0}$, μ is the linear attenuation coefficient, and d is the thickness of the absorber.

Simulations were made in MCNP5 environment (Monte Carlo code group) with the assumption that the flow of photons has a laminar character. This can be true in practice, if detectors are positioned closely enough to the material.

BLL allowed gaining better temporal resolution of a radiation gauge if compared with classical methods; as a result, improved resolution of this method eliminates the need for a stronger radioactive source.

## 3 Limitations

As mentioned about limitations of the Bouguer’s law in Sec. 2.1, there are several aspects that must be taken into account when using BLL. Some of the limitations are not specifically related to BLL but are rather general limitations in the tissue optics assumptions. In this section we will discuss the main physical effects limiting applicability of BLL and some possible solutions to overcome these obstacles.

### 3.1 Scattering Effects

Scattering is the main problem for adequate use of the BLL; if the scattering role is not negligible, the chromophore concentration calculations are inaccurate.^26^ In the biological tissues, scattering occurs due to turbidity or the suspended micro-particles.^30^ Efficiency of the Mie and Rayleigh scattering, both taking place in tissues, depends on the light wavelength.^31^ In particular, the OPL in a scattering tissue is wavelength dependent and therefore influences absorption.^5^

The impacts of absorption and scattering can be measured separately by time-resolved spectroscopy or intensity-modulated spectroscopy, therefore the scattering cross-talk can be estimated and BLL still be used. One should note, however, that light scattering in the tissues changes during muscle activation due to tension kinetics^32^ and during neuronal activation in the brain under physiological or pathophysiological conditions.^1^^,^^33^

Sometimes scattering can be assumed to be wavelength-independent if an algorithm with wavelength-dependent OPL is applied.^34^ Accordingly, impact of tissue scattering can be removed from the calculations in this case.

Feder et al.^35^ proposed a measuring method how to exclude the scattering and concentrate only on absorption. Measurements must be made at a self-calibration point, the iso-pathlength (IPL) point in a specific angle from cylindrical tissues with only one wavelength. In this point OPL is constant for different scattering coefficients and light intensity does not depend on scattering:

$$I(\theta_{IPL},\mu_{a})=I_{0}(\theta_{IPL})\exp[-\mu_{a}\cdot d\cdot DPF(\mu_{a})],$$ (25)

where $I(\theta_{IPL},\mu_{a})$ is light intensity after passing through the medium at IPL point, $I_{0}(\theta_{IPL})$ is light intensity without absorption at IPL point, $\mu_{a}$ is the absorption coefficient, and d is physical distance; $d=D\cdot \sin[\frac{180-\theta_{IPL}}{2}]$, where D is the cylinder diameter, θ is the light exit angle, and DPF is the differential pathlength factor.

### 3.2 Concentration Effects

Concentration determined by BLL is related only to the absorbing species, other species don’t affect it even if both kinds of species are in equilibrium.^36^ However, BLL fails at high chromophore concentrations due to absorber–absorber interaction; if changes in the concentrations of absorbers are negligible, BLL works correctly.^37^

Following the electromagnetic theory, particle concentration is proportional to the imaginary part of the mean polarizability. Molar absorption coefficient is a function of the inverse index of refraction, and, therefore, a function of concentration. Refractive index n≈1, if the concentration c is small:

$$n=\sqrt{1+c\frac{N_{A}\cdot \alpha }{\varepsilon_{0}}},$$ (26)

where $N_{A}$ is Avogadro’s constant, α is mean polarizability, and $\varepsilon_{0}$ is vacuum permittivity. Therefore, Beer’s empiric law at small concentrations will work properly.^38^

Some chemical interactions within large concentrations can influence the Beer law accuracy, as well. Tang et al.^39^ analyzed chiral composition with different concentrations. Electronic absorption (UV) and circular dichroism (CD) signals were measured to analyze enantiomeric excess. Linear correlation between concentration and electronic absorption was observed at small sample amounts. However, when sample concentration was 10% or more, deviations from Beer’s law emerged.

Deviations from BLL can be observed in the absorption spectra of phenols and acetamino compounds at high concentrations and even at relatively low concentrations in alcohol solutions.^40^ When ion concentration in dilute solution exceeds 0.01 M, the average distances between particles are small enough for ions to affect the charge distribution thus influencing the light absorbing character of the molecules and the whole solution.^41^

Concentration of absorbers may be distributed non-uniformly over the sampling volume; accordingly c in the BLL equation has to be replaced by the average concentration along the light path. Light intensity decrease depends on the number of light-absorbing particles, independently of their distribution.

### 3.3 Fluorescence Effects

Fluorescence of tissue components is the result of absorption; if both processes are taking place simultaneously, validity of BLL is questionable.

In an experiment reported in Ref. 42, first separately spectra for pure absorbing components, and for an additional fluorescence spectral components, were measured; afterward the spectra were summed taking into account appropriate weightings; then the spectrum of a complete mixture was measured. In result, both spectra differed.

### 3.4 Chemical Equilibria

Nonlinearity can occur when the absorbing species undergoes association, dissociation, or reaction with the solvent creating products having different absorption properties than the analyte. Often this effect occurs with monomer-dimer, acid/base, and metal complexation equilibria with more than one complex, or solvent-analyte association equilibria.^41^ If the analyte concentration is high, refractive index of the solution can change and cause nonlinearity by altering the position, size, or solid angle of the image transmitted to the detector.

Real absorbance with respect to chemical and/or physical effects is non-linear,^43^

$$A_{\text{real}}=C_{1}\cdot c\cdot d+C_{2}\cdot c^{2}\cdot d+\ldots +C_{m}\cdot c^{l}\cdot d,$$ (27)

where c is concentration, d is photon pathlength, and $C_{1},C_{2}\ldots C_{m}$ are constants.

### 3.5 Absorbing Packets

Absorbing molecules can be concentrated in packets (clusters), which are distributed through the solution. Such packets are assumed to be non-scattering. Duysens^44^ suggested describing suspension absorbance of packets as

$$A_{\mathrm{sus}}=-\frac{l}{d_{c}}\;\ln(1-\varphi (1-T_{p})).$$ (28)

For the equivalent solution:

$$A_{\mathrm{sol}}=-\frac{d}{d_{c}}\;\ln\;T_{p},$$ (29)

where d is the optical cuvette length, $d_{c}$ is the cubic absorber’s packet side length, $T_{p}$ is transmission across two opposing faces of a single cubic packet ($0\leq T_{p}\leq 1$), φ is fractional volume – density of the packet (0≤φ≤1).

### 3.6 Integrated Absorbance

Planck^45^ used dispersion theory and proved that if absorption is high, then BLL does not hold due to local field effects. It works only for spectrally narrow and weak absorption bands and the absorption maximum shifts with an increasing concentration of oscillators.

If local field effects are insignificant, the index of refraction and the dielectric constant are considered to be complex functions of the frequency and dependence of the concentration related to BLL can be found. Absorbance values at certain wavenumbers vary nonlinearly with concentration.

Mayerhöfer et al.^13^ proved that there is a linear relation between the integrated absorbance and the concentration. Therefore, transition probability does not depend on concentration. Normalized integrated absorbance can be expressed as

$$A_{0}=\frac{2\pi }{\ln\;10}\;\underset{c\to 0}{\lim}(k(c,{\overset{˜}{\nu }}_{0})\cdot \frac{1}{c})\frac{\int_{{\overset{˜}{\nu }}_{a}}^{{\overset{˜}{\nu }}_{b}}\overset{˜}{\nu }k(c,\overset{˜}{\nu })d\overset{˜}{\nu }}{\int_{{\overset{˜}{\nu }}_{a}}^{{\overset{˜}{\nu }}_{b}}\overset{˜}{\nu }k(c=1\;mol/1,\overset{˜}{\nu })d\overset{˜}{\nu }},$$ (30)

where $k(\overset{˜}{\nu })=\sqrt{\frac{\sqrt{\varepsilon_{r}^{\prime }{\left(\overset{˜}{\nu }\right)}^{2}+\varepsilon_{r}^{\prime \prime }{\left(\overset{˜}{\nu }\right)}^{2}}-\varepsilon_{r}^{\prime }(\overset{˜}{\nu })}{2}}$ is the index of absorption, $\varepsilon_{r}(\tilde{\nu })=1+c\frac{S^{*2}}{{\tilde{\nu }}_{0}^{2}-{\tilde{\nu }}^{2}-i\tilde{\nu }\gamma }$ is the relative dielectric function, c is molar concentration, $S^{*2}$ is molar oscillator strength, ${\tilde{\nu }}_{0}$ is the oscillator position, and γ is the damping constant.

If sum rule is used, then the number of oscillators, bands, and their shapes does not affect the results; furthermore, they even can spectrally overlap:

$$\int_{0}^{\infty }\overset{˜}{\nu }k(\overset{˜}{\nu })d\overset{˜}{\nu }=\frac{\pi }{2}S^{2}=\frac{\pi }{4}c\cdot S^{*2}.$$ (31)

As limitation, medium must be isotropic and perfectly homogenous; for layered and/or anisotropic materials it does not hold. The electric field intensity inside the medium can change only due to absorption. There are no local interference effects, scattering, plasmonic enhancement, or electromagnetic coupling.

### 3.7 Index of Absorption

Attenuated total reflection technique clearly states that there are some deviations from BLL.^46^ It is applicable only for weak absorptions, when index of absorption (defined in Sec. 3.6) is smaller than 0.1. Despite the fact that organic and biological substances have relatively low absorption, they may have some bands that are much stronger, even 2.5 times higher than the limit.^38^

### 3.8 Anisotropy Effects

Most biologically important molecules or unit cells are optically anisotropic. Probability of absorption is zero when the electric vector is perpendicular to the optical axis and at maximum when the angle is zero. There are no unusual optical properties if the light is unpolarized. In case of plane polarized light, the extinction varies with orientation of the plane of polarization. As a consequence, absorbance is not linearly related to concentration.^14^

In case of anisotropy in the gas molecules, linear dichroism theory can be applied.^47^ In this case absorption is determined only by the angle between transition moment and light polarization.

### 3.9 High Reflectance of Sample Medium

If absorbance is measured from the transmission spectrum and the medium is highly reflective, light intensity is lost also due to reflectance on boundaries and absorbance shows falsely higher values. Measured transmittance spectrum shows a wave-like baseline, which represents interference fringes. One reason for this could be that light multiple times is reflected from one surface of the slab and then from the other one.^38^ BLL works better for media with lower reflection.^30^

### 3.10 Interference Effects

Light wave amplitude can change due to constructive or destructive interference. Part of propagating light wave is transmitted through a slab, but another part can be reflected at the boundary between the layer and exit medium. The reflected part and the forward traveling part interfere; as a result, the electric field intensity changes within the slab depending on the location, wavelength, and the slab thickness. A standing wave situation can occur even if light source is non-coherent and the layer is not very thin. This leads to a conclusion that absorbance does not depend linearly on the slab thickness. Position of the absorption maximum in the spectrum can be shifted, as well.^38^

### 3.11 Spectral Bandwidth Effects

BLL is strictly valid only for monochromatic radiation. In practice, the light source always has a finite bandwidth—even concerning lasers, spectral line sources, and monochromator-selected light. The measured transmission can be corrected using the following expression:^48^

$$\overset{˜}{T}=\frac{\int_{\lambda_{1}}^{\lambda_{2}}I(\lambda )S(\lambda )10^{-A(\lambda )}d\lambda }{\int_{\lambda_{1}}^{\lambda_{2}}I(\lambda )S(\lambda )d\lambda }.$$ (32)

The measured attenuation:

$$A^{\prime }=\mathrm{lg}\;\frac{1}{\overset{˜}{T}},$$ (33)

where I(λ) in Eq. 32 is light input intensity, S(λ) is detector sensitivity, and A(λ) is monochromatic absorbance; for example,

$$A(\lambda )=\frac{\Delta A^{\prime }}{\Delta \lambda }(\lambda -\overset{¯}{\lambda })+\overset{¯}{A},$$ (34)

where $\Delta A^{\prime }$ is the true attenuation difference between $\Delta \lambda =\lambda_{2}-\lambda_{1}$, and $\overset{¯}{A}$ is the theoretically expected value for the mean of A(λ) at $\overset{¯}{\lambda }$.

Greater departures from BLL linearity can be expected with increasing differences between molar absorptivity $\varepsilon^{\prime }$ and $\varepsilon^{\prime \prime }$ related to two different wavelengths $\lambda^{\prime }$ and $\lambda^{\prime \prime }$. The measured absorbance:

$$A_{m}=\log(\frac{P_{0}^{\prime }+P_{0}^{\prime \prime }}{P_{0}^{\prime }10^{-\varepsilon^{\prime }lc}+P_{0}^{\prime \prime 10^{-\varepsilon^{\prime \prime }lc}}}),$$ (35)

where $P_{0}^{\prime }$ and $P_{0}^{\prime \prime }$ are incident power at $\lambda^{\prime }$ and $\lambda^{\prime \prime }$.

If the wavelength region is selected in which the molar absorptivity of the analyte is approximately constant, BLL can be used correctly. Absorption bands of tissues in the UV region usually are narrower than those in the visible region, therefore, it is best to select a wavelength band near the wavelength of maximum absorption where the analyte absorptivity changes little with wavelength.^41^

### 3.12 Stray Radiation Effects

Stray radiation or stray light occurs when wavelengths other than those related to the selected spectral band reach the detector, for example, from scattering and reflection off lenses, mirrors, filters, or gratings. This effect flattens the absorption peak. The measured transmittance will appear higher than it should be because the stray radiation is not absorbed by the medium. The observed absorbance will be lower than the true absorbance,^41^

$$A=\log(\frac{P_{0}+P_{s}}{P+P_{s}}),$$ (36)

where $P_{0}$ is radiant power, P is transmitted power, and $P_{s}$ is radiant power of the stray light.

As the concentration increases, this effect becomes more important and the transmittance can be mostly dependent on the stray radiation. To select the appropriate wavelength bands, double or triple monochromators can be used. Light from the source that reaches the detector without passing the medium certainly causes inaccuracies.^48^

However, there are some simple solutions for this problem, e.g., by using polarizers to eliminate stray light and glare. It also improves sharpness of the image without significantly reducing optical efficiency and inhomogeneities comparing with the original image without polarizer.^49^

### 3.13 Dichroism Effects

Substances such as tissues may have different absorption spectra for two different polarizations of light—the effect called dichroism. This effect has been observed for the nucleic acids in cells. Fortunately, their concentration in tissue is small and does not change rapidly with time, therefore this effect can be assessed as negligible.^48^

### 3.14 Instrumental Effects

Instrumental effects are, for example, finite spectral resolution, non-linear detector response to light intensity changes or inappropriate spectral slit width that affects peak height for sharp absorption bands.^43^

If instrumental effects do not affect the measurement, then measured absorbance A is the same as real absorbance $A_{\text{real}}$:

$$A=A_{\text{real}}=a\cdot c\cdot d,$$ (37)

where c is concentration, d is slab thickness, a is constant.

However, if there is any instrumental caused inaccuracy, then the real absorbance is not recorded by the instrument. Regardless the measured absorbance A can be described in the first approximation by the real absorbance $A_{\text{real}}$:

$$A=A_{\text{real}}+pA_{\text{real}}+q{A_{\text{real}}}^{2}+\ldots +z{A_{\text{real}}}^{n},$$ (38)

where p,q,…,z are constants and $A_{\text{real}}=C_{1}\cdot c\cdot d+C_{2}\cdot c^{2}\cdot d+\ldots +C_{m}\cdot c^{l}\cdot d$, $C_{1},C_{2}\ldots C_{m}$ are constant.

## 4 Application Examples of BLL

### 4.1 Determination of Blood Oxygen Saturation

Anderson et al.^19^ proposed use of the Beer–Lambert law to calculate blood oxygen saturation, if measurements are taken at two spectrally close isosbestic points (520 and 546 nm).^50^ In this case, concentration and light pathlength stay about the same and also scattering is approximately the same. Therefore, it is possible to calculate the scattering parameter B:

$$B=\frac{(\varepsilon_{1}/\varepsilon_{2})OD_{2}-OD_{1}}{(\varepsilon_{1}/\varepsilon_{2})-1},$$ (39)

where $\varepsilon_{1}/\varepsilon_{2}$ is the ratio of molar extinction coefficients for a hemoglobin solution at two isosbestic wavelengths ($\lambda_{1}$ and $\lambda_{2}$), OD is optical density.

If optical density at a wavelength $\lambda_{M}$ close to both isobestic points (e.g., 555 nm) is measured, blood oxygen saturation can be calculated:

$$SO_{2}=m(\frac{OD_{M}-B}{OD_{I}-B})+b,$$ (40)

where m and b are constants, which are determined from extinction coefficients for oxyhemoglobin $\varepsilon_{O_{2}Hb}$ and deoxyhemoglobin $\varepsilon_{HHb}$ at $\lambda_{I}$ and $\lambda_{M}$; $OD_{I}$ is optical density at the isosbestic point $\lambda_{I}$, where $\varepsilon_{HHb}=\varepsilon_{O_{2}Hb}$; and $OD_{M}$ is the optical density at $\lambda_{M}$, where $\varepsilon_{O_{2}Hb}$ and $\varepsilon_{HHb}$ are considerably different.

### 4.2 Determination of Cerebral Tissue Oxygenation

One of applications for MBLL is to measure the cerebral tissue oxygenation index via forehead skin and to calculate three components: $\Delta O_{2}Hb$ is oxyhemoglobin, ΔHHb is deoxyhemoglobin, and ΔCtOx is the redox state of cytochrome oxidase in the brain. Method was implemented in NIRO-300 – tissue clinical monitoring device developed by Suzuki et al.^51^ Measurements were taken at four wavelengths: 775, 810, 850, and 905 nm.

Ong et al.^3^ used the program TracePro^52^ to model light propagation in one layer medium and compared these results with MBLL data to find bilirubin concentration ($c_{bil}$), oxygen saturation ($SO_{2}$), parameters G and d in blood. Measurements were taken at the wavelength band 440 to 500 nm with a 2-nm step, and the following expression was used:

$$OD(\lambda )=G+\varepsilon_{bil}c_{bil}d+((\varepsilon_{O_{2}Hb}-\varepsilon_{HHb})SO_{2}+\varepsilon_{HHb})T,$$ (41)

where T=150  g/l is the total blood concentration.

MBLL then can be expressed in the following form:^53^

$$I=\chi I_{0}e^{-\mu_{a}\cdot DPF\cdot d+G},$$ (42)

where I is detected intensity, χ is the coupling efficiency of light, $I_{0}$ is the intensity of the source, DPF is the differential pathlength factor, d is the distance between source and detector fibers, G is the scattering-dependent geometry factor, and $\mu_{a}$ is the absorption coefficient of the tissues. χ and G are unknown, therefore near infrared spectroscopy (NIRS) commonly is used to measure changes in intensity under conditions where χ and G remains constant.

The main idea of this method is to look at small wavelength differences Δλ:

$$\Delta \;\ln\;I=\Delta \;\ln\;I_{0}-d\Delta \lambda \underset{n}{\sum }c_{n}(DPF\frac{\partial \varepsilon_{n}}{\partial \lambda }+\varepsilon_{n}\frac{\partial DPF}{\partial \lambda }).$$ (43)

Laser diodes can be used for tissue illumination as their differences in wavelength can be a few nanometers and spectral width <1  nm.

Ri et al.^4^ used MBLL and the diffuse scattering model for studies of muscle tissue. They exploited 740- and 850-nm LEDs for illumination and a CCD camera, for image capturing. Scattering coefficient of light in tissues decreases linearly with wavelength in the visible and NIR regions, and the attenuation slopes are almost constant for different tissue types.^51^ Therefore a fixed DPF value can be used.

Mean tissue oxygen saturation value is calculated first in every pixel of the image separately:

$$SO_{2i}=\frac{-\varepsilon_{HHb}(\lambda_{1})+rm_{i}\cdot \varepsilon_{HHb}(\lambda_{2})}{\varepsilon_{O_{2}Hb}(\lambda_{1})-\varepsilon_{HHb}(\lambda_{1})-rm_{i}(\varepsilon_{O_{2}Hb}(\lambda_{2})-\varepsilon_{HHb}(\lambda_{2}))},$$ (44)

where $O_{2}Hb$ is oxyhemoglobin, HHb is deoxyhemoglobin, ε is the molar absorption coefficient, $rm_{i}=\frac{m_{i}(\lambda_{1})}{m_{i}(\lambda_{2})}$, $m_{i}=(\varepsilon_{HHb}(\lambda_{i})c_{HHb_{i}}+\varepsilon_{O_{2}Hb}(\lambda_{i})c_{O_{2}Hb_{i}})\cdot DPF$, and c is concentration. Afterward, the oxygen saturation map can be constructed.

Izzetoglu and Holtzer^54^ have measured oxy- and deoxy-haemoglobin changes in brain–cortical activity, using diffusely reflected light intensity. 730- and 850-nm LEDs were used as light sources. Two DPF function approaches were chosen for comparison: constant six, which is the most often used value, and a formula for human forehead measurements, proposed by Scholkmann and Wolf.^23^ Also different molar extinction coefficient (ε) values were compared from three different authors: Prahl,^55^ Cope,^48^ and Zijlstra.^56^ Results showed that depending on which values they choose, results differed from 5% to 25%.

MBLL can also be used for diffuse correlation spectroscopy (DCS).^57^ DCS-MBLL for homogeneous tissue can be expressed as

$$\Delta OD_{DCS}(\tau ,d)=-\log(\frac{g_{2}(\tau ,d)-1}{g_{2}^{0}(\tau ,d)-1})\approx d_{F}(\tau ,d)\Delta F+d_{a}(\tau ,d)\Delta \mu_{a}+d_{s}^{\prime (\tau ,d)}\Delta {\mu_{s}}^{\prime },$$ (45)

where $\mu_{a}$ is the absorption coefficient; ${\mu_{s}}^{\prime }$ is the reduced scattering coefficient; d is the source–detector separation, τ is delay-time; $g_{2}$ is the intensity autocorrelation function; F is the blood flow index; and $d_{F}$, $d_{a}$, ${d_{s}}^{\prime }$ are weighting factors.

Equations for two-layer model and heterogeneous tissue can be expressed, as well; this approach could be useful for real-time blood flow monitoring.

### 4.3 Applications for Skin Diagnostics

In the study of Huong et al.,^27^ the object of interest was a double-layer skin model: epidermis – infinite slab without blood; dermis – blood-rich semi-infinitive bottom layer. Subcutaneous layer was not taken into account as light penetrates only up to 2 mm in depth. Monte-Carlo simulations of the multispectral system and skin model were used to determine the maximum penetration depth.^58^ Reduced scattering coefficient ${\mu_{s}}^{\prime }$ was considered the same in both layers.

To find oxygen saturation $SO_{2}$ and carbon monoxide saturation *SCO* values, attenuation data was calculated from MBLL and EMLB and compared with simulated attenuation data from reflected light Monte Carlo simulations.

Another comparison was made with data from experiment: diffuse reflectance measurements were taken from the white ($I_{W}$) and dark ($I_{D}$) references and skin ($I_{S}$).^2^ Light attenuation:

$$A^{\prime }(\lambda )=\log(\frac{I_{W}(\lambda )-I_{D}(\lambda )}{I_{S}(\lambda )-I_{D}(\lambda )}).$$ (46)

Using optimization fitting function fminsearch (MATLAB), values $G_{0}$, $d_{0}$, $G_{1}$, $d_{1}$, and $SO_{2}$ were calculated by comparing the measured attenuation values with the EMLB model.^27^ In this way relative concentration values were acquired instead of absolute. Good results were obtained from the spectral ranges 520 to 645 nm and 530 to 570 nm, at 5-nm spectral resolution.

Spigulis et al.^59^ used BLL for skin tissue chromophore mapping. Relative values of chromophore concentrations were calculated from a set of skin spectral images. Three spectral images (at 448, 532, and 659 nm) were used for relative concentration estimation of melanin, oxyhemoglobin, and deoxyhemoglobin:

$$\left\{ \begin{array}{c} \ln(\frac{I_{1}}{I_{01}})=-d_{1}(\Delta c_{a}\cdot \varepsilon_{a}(\lambda_{1})+\Delta c_{b}\cdot \varepsilon_{b}(\lambda_{1})+\Delta c_{c}\cdot \varepsilon_{c}(\lambda_{1}))\\ \ln(\frac{I_{2}}{I_{02}})=-d_{2}(\Delta c_{a}\cdot \varepsilon_{a}(\lambda_{2})+\Delta c_{b}\cdot \varepsilon_{b}(\lambda_{2})+\Delta c_{c}\cdot \varepsilon_{c}(\lambda_{2}))\\ \ln(\frac{I_{3}}{I_{03}})=-d_{3}(\Delta c_{a}\cdot \varepsilon_{a}(\lambda_{3})+\Delta c_{b}\cdot \varepsilon_{b}(\lambda_{3})+\Delta c_{c}\cdot \varepsilon_{c}(\lambda_{3})) \end{array},\;\right.$$ (47)

where $\varepsilon_{i}(\lambda_{j})$ represents the extinction coefficients of three chromophores (i=a,b,c) at three exploited wavelengths (j=1, 2,3), $d_{j}$ is the light pathlength in skin at a particular wavelength, and $\Delta c_{i}$ is the concentration increase or decrease of chromophore comparing with healthy skin. Application of expression Eq. (47) for all image pixels or pixel groups allowed mapping the chromophore concentration changes in pigmented and vascular skin malformations.

Välisuo et al.^60^ suggested a method to create a linear model for human skin chromophore concentration mapping from spectral images based on the differential BLL. Concentrations of blood and melanin in tissue, as well as oxygen saturation levels were calculated. Results were comparable with data from Monte Carlo multilayer simulation and a spectrometer measurement.

## 5 Discussion and Summary

As demonstrated above, there are lots of specific limitations and assumptions to be taken into account when using the BLL. In most tissue measurements, the basic assumptions of BLL (the incident radiation is collimated, monochromatic, orthogonal to the sample surface, sample is homogeneous and non-scattering, absorbers act independently of each other) are not fulfilled. In addition, BLL can become nonlinear if concentration, index of refraction, or absorbance are too high; effects leading to mistaken results of tissue measurements include anisotropy, scattering, fluorescence, chemical equilibria, interference, dichroism, spectral bandwidth disagreements, stray radiation, and instrumental effects. Eventual impacts of all these factors should be carefully considered when results of tissue measurements are interpreted by means of BLL.

Nevertheless, BLL can be adequately used with additional calculations. The modified BLL accounting for the role of scattering in several ways appears to be a successful approach. The EMBLL and BLL in the time domain form could provide more accurate results, but this requires more time resources to be spent.

We believe that this review of BLL may be helpful for many researchers and engineers dealing with experimental measurements and practical applications of bio-tissue optical properties.

## References

[1] Obrig H. Villringer A., “Beyond the visible—imaging the human brain with light,” J. Cereb. Blood Flow Metab. 23(1), 1–18 (2003). 10.1097/01.WCB.0000043472.45775.29 12500086

[2] Huong A. Tay K. G. Ngu X., “Towards skin tissue oxygen monitoring: an investigation of optimal visible spectral range and minimal spectral resolution,” Univers. J. Electr. Electron. Eng. 6(5), 49–54 (2019). 10.13189/ujeee.2019.061607

[3] Ong P. E. et al., “Modified Lambert Beer for bilirubin concentration and blood oxygen saturation prediction,” Int. J. Adv. Intell. Inf. 5(2), 113–122 (2019). 10.26555/ijain.v5i2.363

[4] Ri Y. U. et al., “Estimation of the hemoglobin concentration and the anatomic structure of muscle by analyzing the near infrared scattering images,” Biomed. Signal Process. Control 61, 102058 (2020). 10.1016/j.bspc.2020.102058

[5] Delpy D. T. et al., “Estimation of optical pathlength through tissue from direct time of flight measurement,” Phys. Med. Biol. 33(12), 1433–1442 (1988). PHMBA7 0031-9155 10.1088/0031-9155/33/12/008 3237772

[6] Contini D. Martelli F. Zaccanti G., “Photon migration through a turbid slab described by a model based on diffusion approximation. I. Theory,” Appl. Opt. 36(19), 4587 (1997). APOPAI 0003-6935 10.1364/AO.36.004587 18259254

[7] Kienle A. et al., “Improved solutions of the steady-state and the time-resolved diffusion equations for reflectance from semi-infinite turbid medium,” J. Opt. Soc. Am. 14, 246–54 (1997). JOSAAH 0030-3941 10.1364/JOSAA.14.000246 8988618

[8] Hiraoka M. et al., “A Monte Carlo investigation of optical pathlength in inhomogeneous tissue and its application to near-infrared spectroscopy,” Phys. Med. Biol. 38, 1859–1876 (1993). PHMBA7 0031-9155 10.1088/0031-9155/38/12/011 8108489

[r9] Prahl S. A., “A Monte Carlo model of light propagation in tissue,” Proc. SPIE 10305, 1030509 (1989). 10.1117/12.2283590

[10] Meglinski I. V. Matcher S. J., “Quantitative assessment of skin layers absorption and skin reflectance spectra simulation in the visible and near-infrared spectral regions,” Physiol. Meas. 23(4), 741–753 (2002). PMEAE3 0967-3334 10.1088/0967-3334/23/4/312 12450273

[11] Saiko G. et al., “Hyperspectral imaging in wound care: a systematic review,” Int. Wound J. 17(6), 1840–1856 (2020). 10.1111/iwj.13474 32830443 PMC7949456

[12] Karamavuş Y. Özkan M., “Newborn jaundice determination by reflectance spectroscopy using multiple polynomial regression, neural network, and support vector regression,” Biomed. Signal Process. Control 51, 253–263 (2019). 10.1016/j.bspc.2019.01.019

[13] Mayerhöfer T. G. Pipa A. V. Popp J., “Beer’s law-why integrated absorbance depends linearly on concentration,” ChemPhysChem 20(21), 2748–2753 (2019). CPCHFT 1439-4235 10.1002/cphc.201900787 31544999 PMC6899465

[14] Commoner B. Lipkin D., “The application of the Beer–Lambert law to optically anisotropic systems,” Science (80-.) 110(2845), 41–43 (1949). 10.1126/science.110.2845.41-a 17735370

[15] Bouguer P., “Essai d’optique sur la gradation de la lumière,” 1729, https://www.amazon.com/Essai-DOptique-Gradation-Lumiere-French/dp/116603044X.

[16] Lambert J. H., “Photometria sive de mensura et gradibus luminis, colorum et umbrae,” 1760, https://www.amazon.com/Photometrie-Photometria-Mensura-Gradibus-Luminis/dp/1279983108.

[17] Beer A., “Bestimmung der Absorption des rothen Lichts in farbigen Flüssigkeiten,” Ann. Phys. 162, 78–88 (1852). 10.1002/andp.18521620505

[18] Twersky V., “Multiple scattering of waves and optical phenomena,” J. Opt. Soc. Am. 52(2), 145–171 (1962). 10.1364/JOSA.52.000145 13923335

[19] Anderson N. M. Paul Sekelj, “Light-absorbing and scattering properties of nonhaemolysed blood,” Phys. Med. Biol. 12(2), 173–184 (1967). PHMBA7 0031-9155 10.1088/0031-9155/12/2/303 6033356

[20] Feder I. et al., “The influence of the blood vessel diameter on the full scattering profile from cylindrical tissues: experimental evidence for the shielding effect,” J. Biophotonics 9(10), 1001–1008 (2016). 10.1002/jbio.201500218 26663658

[21] van der Zee P. et al., “Experimentally measured optical pathlengths for the adult head, calf and forearm and the head of the newborn infant as a function of inter optode spacing,” Adv. Exp. Med. Biol. 316, 143–153 (1992). AEMBAP 0065-2598 10.1007/978-1-4615-3404-4_17 1288074

[22] Fantini S. et al., “Non-invasive optical monitoring of the newborn piglet brain using continuous-wave and frequency-domain spectroscopy,” Phys. Med. Biol. 44(6), 1543–1563 (1999). PHMBA7 0031-9155 10.1088/0031-9155/44/6/308 10498522

[23] Scholkmann F. Wolf M., “General equation for the differential pathlength factor of the frontal human head depending on wavelength and age,” J. Biomed. Opt. 18(10), 105004 (2013). JBOPFO 1083-3668 10.1117/1.JBO.18.10.105004 24121731

[24] Tsuchiya Y., “Photon path distribution and optical responses of turbid media: theoretical analysis based on the microscopic Beer–Lambert law,” Phys. Med. Biol. 46(8), 2067–2084 (2001). PHMBA7 0031-9155 10.1088/0031-9155/46/8/303 11512611

[25] Sassaroli A. Fantini S., “Comment on the modified Beer–Lambert law for scattering media,” Phys. Med. Biol. 49(14), N255–N257 (2004). PHMBA7 0031-9155 10.1088/0031-9155/49/14/N07 15357206

[26] Kocsis L. Herman P. Eke A., “The modified Beer–Lambert law revisited,” Phys. Med. Biol. 51(5), N91–N98 (2006). PHMBA7 0031-9155 10.1088/0031-9155/51/5/N02 16481677

[27] Huong A. Ngu X., “The application of extended modified Lambert Beer model for measurement of blood carboxyhemoglobin and oxyhemoglobin saturation,” J. Innov. Opt. Health Sci. 7(3), 1450026 (2014). 10.1142/S1793545814500266

[28] Barbosa R. I. et al., “Analysis of low-level laser transmission at wavelengths 660, 830 and 904 nm in biological tissue samples,” J. Photochem. Photobiol. B Biol. 209(March), 111914 (2020). 10.1016/j.jphotobiol.2020.111914 32516626

[29] Mosorov V., “The Lambert–Beer law in time domain form and its application,” Appl. Radiat. Isot. 128, 1–5 (2017). ARISEF 0969-8043 10.1016/j.apradiso.2017.06.039 28675867

[30] Rose H. E., “Breakdown of the Lambert–Beer law,” Nature 169(4294):287–288 (1952). 10.1038/169287a0

[31] Jacques S. L., “Optical properties of biological tissues: a review,” Phys. Med. Biol. 58(11) (2013). PHMBA7 0031-9155 10.1088/0031-9155/58/11/R37 23666068

[32] Katz G. M. Mozo A. Reuben J. P., “Filament interaction in intact muscle fibers monitored by light scattering,” PNAS 76(9), 4421–4424 (1979). 10.1073/pnas.76.9.4421 291974 PMC411587

[33] Kohl M. et al., “Separation of changes in light scattering and chromophore concentrations during cortical spreading depression in rats,” Opt. Lett. 23(7), 555–557 (1998). OPLEDP 0146-9592 10.1364/OL.23.000555 18084575

[34] Sato C. Nemoto M. Tamura M., “Reassessment of activity-related optical signals in somatosensory cortex by an algorithm with wavelength-dependent path length,” Jpn. J. Physiol. 52(3), 301–312 (2002). 10.2170/jjphysiol.52.301 12230807

[35] Feder I. Duadi H. Fixler D., “Single wavelength measurements of absorption coefficients based on iso-pathlength point,” Biomed. Opt. Express 11(10), 5760 (2020). BOEICL 2156-7085 10.1364/BOE.401591 33149984 PMC7587282

[36] Swinehart D. F., “The Beer–Lambert law,” J. Chem. Educ. 39(7), 333–335 (1962). JCEDA8 0021-9584 10.1021/ed039p333

[37] Nomura Y. Hazeki O. Tamura M., “Relationship between time-resolved and non-time-resolved Beer–Lambert law in turbid media,” Phys. Med. Biol. 42(6), 1009–1022 (1997). PHMBA7 0031-9155 10.1088/0031-9155/42/6/002 9194125

[38] Mayerhöfer T. G. Pahlow S. Popp J., “The Bouguer–Beer–Lambert law: shining light on the obscure,” ChemPhysChem 21, 2029–2046 (2020). CPCHFT 1439-4235 10.1002/cphc.202000464 32662939 PMC7540309

[39] Tang Q. et al., “Deviations from Beer’s law in electronic absorption and circular dichroism: Detection for enantiomeric excess analysis,” Chirality 31(7), 492–501 (2019). CHRLEP 1520-636X 10.1002/chir.23072 31111586

[40] Ungnade H. E. Kerr V. Youse E., “Deviations from Beer’s law in the ultraviolet absorption spectra of some organic compounds,” Science 113(2943), 601 (1951). 10.1126/science.113.2943.601.a 14845678

[41] Skoog D. A. et al., Fundamentals of Analytical Chemistry, 9th ed., Brooks/Cole Cengage Learn (2014).

[42] Rhys Williams A. T. Spragg R. A., “Multi-component quantitative analysis of fluorescent mixtures not obeying Beer’s Law,” Analyst 111(2), 201–203 (1986). ANLYAG 0365-4885 10.1039/an9861100201

[43] Buijs K. Maurice M. J., “Some considerations on apparent deviations from lambert-beer’s law,” Anal. Chim. Acta 47(3), 469–474 (1969). ACACAM 0003-2670 10.1016/S0003-2670(01)95647-8

[44] Duyens L. N. M., “The flattering of the absorption spectrum of suspensions, as compared to that of solutions,” Biochim. Biophys. Acta 19, 1–12 (1956). BBACAQ 0006-3002 10.1016/0006-3002(56)90380-8 13304065

[45] Planck M., “Zur elektromagnetischen theorie der selektiven absorption in isotropen nichtleitern,” Sitzungsber. K. Preuss. Akad. Wiss 24 480–498 (1903).

[46] Hansen W. N., “Expanded formulas for attenuated total reflection and the derivation of absorption rules for single and multiple ATR spectrometer cells,” Spectrochim. Acta 21(4), 815–833 (1965). SPACA5 0038-6987 10.1016/0371-1951(65)80039-X

[47] Zbinden R., “Infrared spectroscopy of high polymers,” Science (80-.) 146(3652), 1667–1668 (1964). 10.1126/science.146.3652.1667-a

[48] Cope M., “The development of a near infrared spectroscopy system and its application for non invasive monitory of cerebral blood and tissue oxygenation in the newborn infants,” Diss. Univ., London, p. 342 (1991).

[49] Tong X. et al., “Effect of polarizer on optical efficiency and inhomogeneity of diffractive optical elements,” Optik (Stuttg). 192(June), 162981 (2019). 10.1016/j.ijleo.2019.162981

[50] Pittman R. N. Duling B. R., “A new method for the measurement of percent oxyhemoglobin,” J. Appl. Physiol. 38(2), 315–320. (1975). 10.1152/jappl.1975.38.2.315 1120757

[51] Suzuki S. et al., “Tissue oxygenation monitor using NIR spatially resolved spectroscopy,” Proc. SPIE 3597(July), 582–592 (1999). 10.1117/12.356862

[52] Lambda Research Corporation, “TracePro, illumination and non-imaging optical design & analysis too,” https://www.lambdares.com/tracepro/ (accessed 11 April 2021).

[53] Hebden J. C., “Exploring the feasibility of wavelength modulated near-infrared spectroscopy,” J. Biomed. Opt. 25(11), 110501 (2020). JBOPFO 1083-3668 10.1117/1.JBO.25.11.110501 PMC7610139 33150775

[54] Izzetoglu M. Holtzer R., “Effects of processing methods on fNIRS signals assessed during active walking tasks in older adults,” IEEE Trans. Neural Syst. Rehabil. Eng. 28(3), 699–709 (2020). 10.1109/TNSRE.2020.2970407 32070987 PMC7768789

[55] Prahl S., “Tabulated molar extinction coefficient for hemoglobin in water,” 1998, https://omlc.org/spectra/hemoglobin/summary.html (accessed 6 April 2021).

[56] Zijlstra W. G. Buursma A. van Assendelft O. W., “Visible and near infrared absorption spectra of human and animal haemoglobin: determination and application,” Rancho Cordova, CA, USA: VSP, 2000, https://journals.lww.com/ccmjournal/Citation/2002/01000/Visible_and_Near_Infrared_Absorption_Spectra_of.52.aspx (accessed 6 April 2021).

[57] Baker W. B. et al., “Modified Beer–Lambert law for blood flow,” Biomed. Opt. Express 5(11), 4053 (2014). BOEICL 2156-7085 10.1364/BOE.5.004053 25426330 PMC4242038

[58] Vogel A. et al., “Using noninvasive multispectral imaging to quantitatively assess tissue vasculature,” J. Biomed. Opt. 12(5), 051604 (2007). JBOPFO 1083-3668 10.1117/1.2801718 17994873 PMC2443549

[59] Spigulis J. et al., “Smartphone snapshot mapping of skin chromophores under triple-wavelength laser illumination,” J. Biomed. Opt. 22(9), 091508 (2017). JBOPFO 1083-3668 10.1117/1.JBO.22.9.091508 28253387

[60] Välisuo P. et al., “New closed-form approximation for skin chromophore mapping,” J. Biomed. Opt. 16(4), 046012 (2011). JBOPFO 1083-3668 10.1117/1.3562976 21529081

